# Construction of a management and prevention program for targeted therapy-induced hand-foot skin reaction

**DOI:** 10.3389/fmed.2025.1718588

**Published:** 2026-01-05

**Authors:** Xi Zhou, Lai Wei, Yong Fang, Miao Du, Shubao Jing, Xi Wang, Xiaolang Cao, Ting Li, Liping Dong

**Affiliations:** 1 School of Nursing, Changsha Medical University, Changsha, China; 2 Hunan Maternal and Child Health Care Hospital, Changsha, China; 3 School of International Studies, Sichuan University, Sichuan, China; 4 Department of Nursing, Zhuhai Campus of Zunyi Medical University, Zhuhai, China; 5 Department of Otorhinolaryngology and Head and Neck Surgery, Central Hospital, Loudi, Hunan, China

**Keywords:** evidence-based, hand-foot skin reaction, nursing, prevention, symptom management

## Abstract

**Aims:**

To construct a management and prevention program for targeted therapy-induced hand-foot skin reactions (HFSR).

**Design:**

A systematic review with meta-analysis.

**Methods:**

Based on a literature review and expert consensus meetings, a two-round expert panel discussion involving 10 experts was conducted to finalize the HFSR management and prevention program.

**Data sources:**

Articles were systematically searched on the CNKI, Wanfang Database, VIP, PubMed, UpToDate, Embase, and Cochrane Library, and guideline publication websites, NGC, NCCN, NICE, SIGN and ESMO databases from inception up to June 2023.

**Results:**

The final program comprised 8 primary indicators, 16 secondary indicators, 36 tertiary indicators, and 54 quaternary entries. These include baseline level and hand-foot skin assessment, skin erythema care, skin keratosis care, skin blister management, skin ulcer care, skin pain management, management of other accompanying symptoms, and hand-foot protection and prevention education.

**Conclusion:**

This management and prevention program, constructed based on evidence and expert discussion, is scientifically sound and clinically applicable, providing reliable guidance for the management and prevention of HFSR in clinical practice.

**Impact:**

Based on the best available evidence for managing and preventing hand-foot skin reactions induced by targeted therapies, we recommend that nursing leaders implement personalized symptom management plans tailored to the common symptoms of these reactions. Such an approach may help in the early detection of hand-foot skin reactions and alleviate patients’ skin pain effectively.

## Highlights

- This study is the first to develop the “Evidence-based management plan for hand-foot skin reaction caused by targeted drugs” through evidence-based methods, which is formulated according to the local cultural background and actual clinical situation. Additionally, this plan offers practical guidelines for clinical skin care practices and delivers personalized symptom management strategies for patients with hand-foot skin reactions.
- On the basis of extensive guidelines and consensuses on skin toxicity reactions caused by targeted drugs, the management evidence of hand-foot skin reaction caused by targeted drugs is scientifically and systematically summarized.
- In this study, we classified items according to common symptoms of hand-foot skin reaction. Compared to conventional nursing, this approach is more systematic and comprehensive, making it easier for clinical staff to learn and apply in practice.

## 1 Introduction

Hand-foot skin reaction (HFSR), different from traditional chemotherapy-induced hand-foot syndrome (HFS), refers to skin adverse reactions caused by targeted therapy. It is one of the most severe skin toxicities induced by anticancer drugs (1). The typical clinical feature of HFSR is excessive keratinization of the skin on the hands and feet. Other common clinical manifestations include varying degrees of palmar-plantar numbness, swelling, erythema, and burning pain (2). A meta-analysis of the incidence of targeted therapy-induced HFSR reported a total incidence range of 4.5 to 60.5%, with severe cases (grade ≥3) ranging from 1.8 to 21.6% (3). Dose reduction, drug discontinuation, or interruption of cancer treatment are the primary strategies to alleviate or eliminate the burden of HFSR symptoms, which significantly impact the treatment process and quality of life of cancer patients (4). Clinicians tend to focus more on the efficacy of targeted drugs rather than the skin adverse reactions they cause (5). Studies have shown that early prevention, treatment, and education can reduce the incidence and severity of HFSR (6). However, research on nursing interventions for HFSR patients is limited. Additionally, a comprehensive management program for HFSR is yet to be identified, and existing information is fragmented. Therefore, the present study integrates existing evidence from literature and expert clinical experience to develop a comprehensive management and prevention program for HFSR, to provide a scientific basis for healthcare professionals to implement early education and clinical interventions for this patient group.

## 2 Methods

### 2.1 Formation of the research team

The research team comprised 8 members, including one master’s supervisor in nursing (Master’s, Chief Nurse), two oncology nurse managers (Bachelor’s, Associate Chief Nurses), one wound and stoma specialist nurse (Bachelor’s, Nurse-in-charge), one oncology physician (Ph.D., Associate Chief Physician), two graduate students in oncology nursing, and one clinical graduate student in oncology surgery. The three master’s students and the wound and stoma specialist nurse conducted the literature search and evidence extraction, integration, and evaluation, and provided materials for the expert meetings. The master’s supervisor chaired the meetings and guided the overall program, while other members collaborated on program formulation and discussion.

### 2.2 Literature review

#### 2.2.1 Literature search

The Chinese search terms included “Targeted Therapy/Molecular Targeted Therapy”; “Hand-Foot Skin Reaction/Palmar-Plantar Erythrodysesthesia/Hand-Foot Syndrome/Burgdorf Reaction”; “Symptom Management/Management/Nursing”; and “Clinical Practice Guidelines/Best Practices/Systematic Reviews/Expert Consensus/Randomized Controlled Trials/Quasi-Experimental Studies/Cohort Studies.” The English search terms included “Targeted therapy/molecular targeted therapy”; “Hand-foot skin reaction/hand-foot redness and swelling acral erythema/hand-foot syndrome/Burgdorf reaction”; “Symptom management/management/care”; and “Clinical Practice Guide/Best Practice/System Overview/Expert Consensus/Randomized Controlled Study/Quasi-Experimental Study/Cohort Study.” Relevant databases, China National Knowledge Infrastructure (CNKI), Wanfang Database, VIP Database, PubMed, UpToDate, Embase, and Cochrane Library, and guideline publication websites, including the National Guideline Clearinghouse (NGC), National Comprehensive Cancer Network (NCCN), National Institute for Health and Care Excellence (NICE), Scottish Intercollegiate Guidelines Network (SIGN), and European Society for Medical Oncology (ESMO), were systematically searched using a combination of subject terms and free-text terms.

#### 2.2.2 Inclusion and exclusion criteria

Inclusion criteria: Studies involving cancer patients taking targeted drugs; research content including the prevention and management of HFSR; and publications including guidelines, evidence summaries, systematic reviews, expert consensus, basic clinical research trials, etc. Exclusion criteria: Inability to obtain full texts; low-quality literature; and duplicate literature entries. Priority was given to evidence-based, high-quality, and recent publications.

#### 2.2.3 Quality assessment criteria

The quality of the literature was assessed by two nursing graduate students trained in evidence-based research. Any discrepancies were resolved through consensus discussion with a third party. The Appraisal of Guidelines for Research & Evaluation II (AGREEII) tool was used to assess the quality of guidelines (7); the Measurement Tool to Assess Systematic Reviews 2 (AMSTAR 2) tool was used to evaluate the quality of systematic reviews (8, 9); randomized controlled trials, cohort studies, expert consensus, and opinions were assessed using appropriate tools from the Joanna Briggs Institute (JBI) for Evidence-Based Healthcare (9).

#### 2.2.4 Evidence extraction, integration, and evaluation

The evidence was graded and recommended using the Joanna Briggs Institute (JBI) Levels of Evidence and Grades of Recommendation system (2014). The included evidence was classified according to the type of study design, with levels ranging from 1 to 5, from highest to lowest quality. Based on the clinical significance, feasibility, effectiveness, and appropriateness of the evidence, the strength of recommendations was determined as Grade A (strong recommendation) and Grade B (weak recommendation). After integration and refinement, a preliminary symptom management and prevention plan for targeted therapy-induced HFSR was developed.

### 2.3 Expert meetings

#### 2.3.1 Selection of meeting participants

Experts were selected using purposive sampling. Inclusion criteria for experts were as follows: (1) professionals engaged in cancer treatment and nursing, with rich knowledge and experience in the field; (2) at least 10 years of work experience; (3) holding a bachelor’s degree or higher; (4) having a professional title of intermediate level or above; (5) able to provide valuable and constructive opinions and suggestions, willing to participate in the study, and capable of completing two rounds of high-quality meetings on time.

#### 2.3.2 Expert meeting planning and facilitation

The expert meeting materials were self-designed as follows:

Expert Meeting Notice: A brief introduction to the background and significance of the study, the process, and the purpose of the meeting.

Self-Evaluation Form: Information on the general situation of the experts, their familiarity with the questionnaire content, and their understanding of the field.

Expert Opinion Consultation Form: Experts rated the “feasibility” and “importance” of primary and secondary indicators in the plan. A Likert five-point scale was used, with 1 point indicating “not important/not feasible” and 5 points indicating “very important/very feasible.” Additionally, each indicator had a suggestion box for experts to provide modifications, additions, or deletions to the content.

Meeting Process: During the meeting, a project leader chaired the session, introducing the background and process of the meeting and explaining the source and scoring instructions for each item in the plan. The main consultation content included: reviewing the general framework of the evidence-based symptom management plan for targeted therapy-induced HFSR and determining whether the detailed content of each item needs optimization or correction.

A preliminary management and prevention plan was developed. After two rounds of expert group discussions, the content of the item pool was revised and summarized based on expert opinions, ultimately forming the symptom management plan for targeted therapy-induced HFSR. Researchers guided the experts without subjective or suggestive prompts to evaluate and discuss the feasibility, applicability, and clinical significance of each piece of evidence based on their work experience and clinical application. Another researcher recorded the meeting and took detailed notes on the discussion.

### 2.4 Statistical analysis

The results of the two rounds of expert meetings were organized and analyzed using Excel and SPSS 25.0 software. Quantitative data were expressed as mean ± standard deviation and coefficient of variation, whereas categorical data were presented as frequency and composition ratio. The authority coefficient of the experts (Cr) was determined by the coefficient of judgment basis (Ca) and the coefficient of familiarity (Cs). The Cr was calculated using the following formula: Cr = (Ca + Cs)/2.

## 3 Results

### 3.1 Basic characteristics of included literature

A total of 469 articles were retrieved and imported into Endnote X8 for deduplication, resulting in 56 articles. After screening the article titles, abstracts, and inclusion/exclusion criteria, 15 articles were included in the final analysis, of which 3 were guidelines (10–12), 2 were systematic reviews (13, 14), 1 was best evidence summary, 6 were expert consensus/opinions (15–20), and 3 were randomized controlled trials (21–23). All three guidelines were rated as Grade A based on quality assessment. The two systematic reviews were rated as “no” for items 7 and 8, whereas the remaining items were rated as “yes.” All six items evaluating the authenticity of the expert consensus/opinions were rated as “yes.” Two randomized controlled trials were rated as “no” for item 5; one trial with an “unclear” rating for items 2 and 3, was rated as Grade B quality, whereas the other trial with a “yes” rating for all items, was rated as Grade A. The general characteristics of the included literature are presented in Table 1.

**Table 1:** Basic information of included references.

Inclusion of literature	Source of evidence	Type of literature	Literature topics	Published (year)
Oncology Nursing Professional Committee of China Anti-Cancer Association (12)	CNKI	Guidebook	Practice Guidelines for Cancer Symptom Management in China - Skin Reactions	2019
European Society of Medical Oncology (ESMO) (10)	NICE	Guidebook	Prevention and management of cutaneous virulence associated with anticancer drugs: an ESMO clinical practice guideline	2021
Williams et al. (11)	PubMed	Guidebook	Guidelines for Cancer Treatment-Related Skin Toxicity	
Ding et al. (13)	PubMed	Systematic evaluation	Targeted therapy and chemotherapy-related skin toxicity: a systematic evaluation and meta-analysis	2020
Pandy et al. (14)	PubMed	Systematic evaluation	Prevention strategies for systemic cancer treatment-related hand-foot syndrome/skin reactions: a meta-analysis of randomized controlled trials	2022
Anderson et al. (31)	PubMed	Summary of evidence	Finding evidence-based approaches to prevent and mitigate hand-foot skin reactions (HFSR) caused by multikinase inhibitors (MKIs)	2015
Spanish Society of Dermatology and Venereology and Spanish Society of Medical Oncology (17)	PubMed	Expert consensus	Clinical management of cutaneous adverse events in patients receiving targeted anticancer therapy and immunotherapy	2018
Multidisciplinary Collaborative Group on Gastrointestinal Tumors, Cancer Hospital, Peking Union Medical College, Chinese Academy of Medical Sciences, China (18)	CNKI	Expert consensus	Expert consensus on targeted therapy for hepatocellular carcinoma	2020
Expert Consensus Panel on Management of Adverse Reactions to Targeted Therapy for Advanced Renal Cancer in China (20)	Pulse of Medicine (TCM)	Expert consensus	Expert consensus on the management of adverse reactions to targeted therapy for advanced renal cancer in China	2015
Wood et al. (19)	PubMed	Expert advice	Practical problems in the management of hand and foot skin reactions due to multikinase inhibitors	2010
Cury-Martins et al. (16)	PubMed	Expert advice	Management of dermatologic adverse events arising from cancer therapy: recommendations of an expert panel	2020
Bracarda et al. (15)	PubMed	Expert advice	Early detection, prevention and management of sorafenib-induced cutaneous adverse events—recommendations of the Sorafenib Working Group	2012
Wang et al. (22)	CNKI	A randomized controlled trial	Chinese medicine compound LC09 granule soak combined with urea ointment for the treatment of Hand and Foot Skin Reactions Caused by Multikinase Inhibitors of Anti-tumor Targeted Drugs. A randomized controlled double-blind clinical study on the skin reactions of hand and foot	2020
Zenda et al. (23)	PubMed	A randomized controlled trial	Hydrocolloid dressings as prophylactic use for multi-targeted kinase inhibitor-induced cutaneous reactions of the hands and feet: protocol for a phase 3 randomized, self-controlled study	2020
Lee et al. (21)	PubMed	A randomized Controlled trial	The effect of urea cream on sorafenib-related hand and foot skin reactions in patients with hepatocellular carcinoma: a multicenter, randomized, double-blind controlled study	2020

### 3.2 General information of experts

Ten experts from three tertiary hospitals in Zhejiang Province were enrolled, of whom 2 were chief physicians, 3 were associate chief physicians, 1 was an attending physician, 1 was a director of the nursing department, 2 were head nurses, and 1 was a specialist nurse in wound and stoma care. The average age was 42.5 ± 5.43 years, ranging from 33 to 52 years. The average years of work experience was 17.8 ± 7.31, ranging from 13 to 31 years. Among them, 3, 6, and 1 held bachelor’s, master’s, and doctoral degrees, respectively. The response rate to the pre-meeting emails was 100%, and the attendance rate at formal meetings was 100%. All experts provided modification suggestions, demonstrating high enthusiasm. The Ca, Cs, and Cr of the two rounds of expert consultation were 0.9125, 0.825, and 0.866, respectively. This confirms a high level of expert authority, supporting the credibility and reliability of the results.

### 3.3 Suggestions from participating experts on the draft plan

During the meetings, all experts actively participated in the discussions. The experts highlighted the symptom management and prevention plan for HFSR should align with the actual conditions of HFSR patients and clinical practices in China to enhance its clinical guidance value. The summarized suggestions and opinions are as follows:

#### 3.3.1 Modifications

Throughout the document, replace “podiatrist” and “orthopedist” with “foot clinic doctor” to better reflect the standard practice in most hospitals in China, in accordance with the experts’ recommendations. Moreover, revise the blister treatment from a 0.2% benzalkonium chloride solution to povidone-iodine disinfection.

Replace quality of life assessment scales Skindex or Dermatology Life Quality Index with the latest HFSR quality of life assessment scale HF-QoL, adopting the expert’s suggestion.

Change use systemic analgesics (e.g., NSAIDs, codeine, pregabalin, *β*-aminobutyric acid agonists, anesthetics) and assess bleeding and renal function before use” to “if local drug control is ineffective, systemic analgesics mainly include celecoxib, and if pain intensifies, codeine, pregabalin, or tramadol may be added as appropriate, with liver and kidney function assessed before use.

Replace soak feet in magnesium sulfate to relieve pressure pain with use magnesium sulfate cold spray for local pain relief.

#### 3.3.2 Additions

“Use flurbiprofen gel patches (Babu patches) for pain relief.”

In managing inflammation associated with infection, it is essential to first assess the infection status. If a local infection is present, topical antibiotics such as erythromycin or penicillin ointment should be applied. In cases where systemic infection signs, such as fever, are observed, appropriate oral or intravenous antibiotics should be administered.

To manage unbroken blisters, add small blisters that do not require treatment. Consolidate desquamation, peeling, exudation, bleeding, and inflammation with infection into the management of other accompanying symptoms as a primary indicator.

Recommend psychological care and dietary guidance. Specifically, healthcare professionals should provide psychological counseling and support and, guide patients should avoid spicy and irritating foods, which are common clinical guidance contents for such patients, adopting the experts suggestion.

#### 3.3.3 Deletions

1. Remove: “Apply glycolic salicylic acid exfoliating cream to the hyperkeratotic areas of the soles at night to keep the skin soft” as it is repetitive with another entry.
2. Remove: In the oral medication intervention section, delete “use low to moderate dose analgesics; apply sunscreen spray to affected areas when needed.”
3. Remove: In the footwear recommendations, delete “apply sunscreen to the feet.” This does not align with our national conditions, based on expert recommendations.

#### 3.3.4 Other suggestions

1. Suggestion: Move the primary entry “Hand and Foot Protection Education” under “Baseline and Hand-Foot Skin Assessment” to emphasize the importance of health education for HFSR prevention, based on expert recommendations.
2. Suggestion: Classify the primary entries according to common HFSR symptoms rather than HFSR grading. This will enable clinical practitioners to perform targeted nursing interventions based on the patient’s actual symptoms, adopting the experts’ suggestions.

#### 3.3.5 Consolidation of expert opinions

- Integrated three expert opinions.
- Merged six entries.
- Deleted 11 entries or redundant content within entries.
- Added eight entries.
- Adjusted the names of eight indicators.
- Proposed three structural adjustments to the plan.
- Ultimately, formed a 52-item management and prevention plan for targeted therapy-induced HFSR symptoms, of which 16 items were classified as Grade A and the remaining as Grade B (Table 2).

**Table 2:** Evidence-based management strategy construction for hand-foot skin reactions induced by targeted drugs.

Level 1 entries	Secondary entries	Tertiary entries	Level 4 entries	Hierarchy of evidence	Recommended level
1 Self-baseline and hand and foot skin assessment	1.1 Self-assessment	1.1.1 Assessment of risk factors	1.1.1.1 Assess the presence of predisposing factors and assess the patient’s occupational, amateur or sports-related hand and foot skin	3	A
		1.1.2 Quality of life assessment	1.1.2.1 Perform full body examination and assessment using quality of life related scales (using established instruments such as the HF-QoL)	5	A
	1.2 Skin assessment of hand and foot	1.2.1 Assessment of the underlying lesions of the hand and foot	1.2.1.1 Assessment of the patient’s underlying hand and foot pathology (e.g., diabetic foot, eczema on the hands and feet, corns, calluses, and fungal disease on the dorsal and lateral aspects of the foot) by a dermatologist or nurse practitioner	5	A
		1.2.2 HFSR grading definition assessment	1.2.2.1 HFSR assessment by healthcare professionals using Common Terminology Criteria for Adverse EventsV5.0 (CTCAEV5.0)	5	A
	1.3 Frequency of assessment	1.3.1 Baseline/treatment assessment	1.3.1.1 During the first two cycles of treatment, healthcare professionals conduct weekly skin assessments of the patient’s self and hands and feet; every 4 weeks from cycle 3; and preemptively address assessed high-risk factors in a timely manner	3	B
2 Hand-foot protection education for prevention	2.1 Timing and importance	2.1.1 Timing of the mission	2.1.1.1 Healthcare professionals educate patients attending the clinic about HFSR prevention and adherence to management strategies within 2–4 weeks prior to treatment	5	B
		2.1.2 Importance of contact with physicians	2.1.2.1 Patients should maintain frequent contact with their physicians to ensure early diagnosis of HFSR	5	A
	2.2 Guidance on the content of prevention	2.2.1 Symptoms, medication notification	2.2.1.1 Healthcare workers carefully inform about the symptoms associated with skin reactions of the hands and feet, and protect tenderness areas, pressure points and pressure-sensitive areas of the hands and feet	5	A
			2.2.1.2 Nursing staff should follow the principles of premedication education, close examination and focusing attention during medication; if the skin condition worsens, consult a specialized department	5	A
		2.2.2 Physical prevention	2.2.2.1 Avoid strenuous exercise or activities that place undue stress on the hands and feet (e.g., lifting weights or walking long distances) for four weeks prior to treatment; reduce excessive friction on the hands and feet (e.g., wearing excessively tight clothing/shoes/gloves).	3	A
		2.2.3 Chemical prevention	2.2.3.1 Avoid contact with alcohol, skin-irritating solvents or disinfectants; avoid the use of common soaps; soak hands and feet in 1:1 vinegar and water for 10 min daily	5	B
		2.2.4 Skin moisturizing care	2.2.4.1 Wash hands and feet with a non-foaming cleanser; apply petroleum jelly-free zinc oxide and magnesium silicate slow-release cream or 10–20% urea cream	1	B
	2.3 Content of the mission	2.3.1 Dietary guidance	2.3.1.1 Instruct patients to avoid spicy and irritating foods	5	B
		2.3.2 Psychological care	2.3.2.1 Psychological guidance and professional support from healthcare professionals	5	B
		2.3.3 Guidance on footwear recommendations	2.3.3.1 Wear loose-fitting shoes or slippers; place gel padding or soft, comfortable, well-fitting insoles in shoes to reduce pressure and friction	5	A
			2.3.3.2 Recommend sandals to alleviate sore spots, and instruct patients to avoid overexposure to sunlight	5	B
			2.3.3.3 Use comfortable shoes that do not exert pressure on the foot, preferably with latex insoles to reduce plantar pressure	5	B
		2.3.4 Hosiery Recommendation Guide	2.3.4.1 Socks should fit well and those that have seams and patches that cause friction or pressure should not be worn	5	B
			2.3.4.2 Multi-layer running socks that reduce sweating help prevent blister formation	5	B
		2.3.5 Recommendations for daily necessities	2.3.5.1 For patients with occupational, amateur, or sports-related calluses, consideration should be given to padding commonly used items (e.g., padding the handles of gardening or carpentry tools, the handles of kitchen utensils, or the handles of sports equipment)	5	B
		2.3.6 Orthotic recommendations	2.3.6.1 Recommendation of custom or semi-custom constructed orthotics for patients with potentially contributing biomechanical abnormalities in foot structure, as identified by an orthopedic surgeon	5	A
3 Skin erythema care	3.1 Assessment and processing	3.1.1 Pre-processing	3.1.1.1 At the first sign of redness/burning, physical labor should be stopped if possible.	5	B
		3.1.2 Topical cream treatment	3.1.2.1 Use of petroleum jelly-free zinc oxide and magnesium silicate palliative creams	2	B
			3.1.2.2 Topical steroids are recommended (e.g., 0.05% fluocinonide cream, hydrocortisone, lumisone, betamethasone propionate, etc.).	1	B
		3.1.3 Instructions for herbal infusions	3.1.3.1 The Chinese medicine compound LC09 granules is recommended for soaking	1	A
4 Skin keratinization care	4.1 Assessment of keratinized sites	4.1.1 Self-monitoring	4.1.1.1 During the first month of targeted therapy, patients should self-examine the skin of their hands and feet daily for new callus formation or deterioration of callus sites	5	A
		4.1.2 Assessment of the degree of angularization	4.1.2.1 In patients with painful or severe cutaneous corns that are not responding to conservative treatment or home care, radiographs may be taken to assess potential contributing biomechanical abnormalities in the foot structure	3	B
			4.1.2.2 For severe cases, referral to a podiatrist or dermatologist is recommended	5	B
	4.2 Treatment of keratinized areas	4.2.1. Pre-processing	4.2.1.1 Repair and removal of the generalized keratinized cortical layer before treatment (avoiding overly aggressive methods of removing the keratinized portion); if the thickening is significant, it may be treated surgically by consulting with a podiatry clinic physician (nurse practitioner or dermatologist)	4	B
			4.2.1.2 Moisturize the skin and control existing plantar and palmar hyperkeratosis	5	A
		4.2.2 Keratinized site inhibition	4.2.2.1 0.1% tazarotene cream and 5% fluorouracil cream recommended	5	B
		4.2.3 Softening of keratinized areas	4.2.3.1 Treat hyperkeratotic areas with topical keratolytic agents (e.g., topical creams or ointments containing 5–10% salicylic acid or 10–40% urea), with the keratotic area being soaked for 5 min and gently dried prior to the application of salicylic acid.	1	A
			4.2.3.2 Apply cumquat ointment and urea ointment, it can be combined with the two drugs in a wet nighttime wrap for mild cases, and a wet nighttime wrap and daytime application for severe cases	5	B
5 Skin blister treatment	5.1 Treatment of blisters	5.1.1 Pre-processing	5.1.1.1 Examine skin blisters on hands and feet; if they are small, treatment can be avoided; if they are large, treat them based on whether they are broken or not.	5	B
		5.1.2 Unbroken treatment	5.1.2.1 Use of topical antibiotics to prevent infection	5	B
			5.1.2.2 Disinfect the area of the blister with povidone-iodine and aspirate the fluid within the blister (using a needle in a disposable syringe) to prevent the growth of bacteria/infection. Insert the needle into the blister and aspirate, or make a small incision with the needle and then squeeze the blister with gauze to remove the fluid	5	B
		5.1.3 Treatment of breakage	5.1.3.1 After the blister has ruptured, treat with a polyethylene glycol-based scar ointment; no bandage is required in the absence of special circumstances	5	B
			5.1.3.2 No treatment is required if the cuticle falls off by itself; if the cuticle covering the blister dries and falls off, it can be removed by cutting the edges	5	B
6 Skin ulcer care	6.1 Treatment of trauma	6.1.1 Cleaning treatment	6.1.1.1 Clean the surface of the ulcer thoroughly, and rehabilitate the new liquid sprayed on the surface of the ulcer, the heavier can be added to recombinant human fibroblast growth factor and insulin	5	B
		6.1.2 Sterilization	6.1.2.1 Decay and ulcers can be treated with disinfectant solutions (e.g., silver sulfadiazine 1%, polyhexane 0.02–0.04%)	5	B
7 Skin pain care	7.1 Localized interventions	7.1.1 Pain intervention at erythema	7.1.1.1 Use of 2% lidocaine gel or 0.05% clobetasol propionate ointment twice daily	2	B
			7.1.1.2 Use of cold compresses/ice packs for pain relief	2	B
		7.1.2 Intervention for pain at keratosis pilaris	7.1.2.1 First assess the thickness of the cuticle on the patient’s hands and feet before selecting a specific intervention	5	A
			7.1.2.2 Analgesic use of flurbiprofen gel paste (babu paste)	5	B
			7.1.2.3 Use magnesium sulfate cold spray for local analgesia	5	B
			7.1.2.4 Apply hydrocolloid dressings containing ceramides to affected areas of the hands and feet	1	B
	7.2 Whole Body Intervention	7.2.1 Internal drug interventions	7.2.1.1 If local medications are not well controlled, systemic analgesics are primarily celecoxib, with codeine, pregabalin, and tramadol added as appropriate if pain worsens, with assessment of hepatic and renal function prior to administration	2	B
8 Management of other concomitant symptoms	8.1 Flaking, peeling	8.1.1 Moisturizing skin treatments	8.1.1.1 Moisturize the skin well with emollient lotion, normally no special treatment is required	5	A
	8.2 Exudate, blood seepage	8.2.1 Wound drying and astringent treatment	8.2.1.1 Keep the skin dry; magnesium sulfate is recommended	5	B
	8.3 Inflammation with infection	8.3.1 Pharmacological interventions	8.3.1.1 Examine the infection and administer topical antibiotics (erythromycin, penicillin ointment) if localized; administer oral or intravenous antibiotics if signs of systemic infection, such as fever, occur	5	B

## 4 Discussion

### 4.1 Clinical significance of the plan

The availability and accessibility of targeted drugs have greatly increased in recent years. The National Healthcare Security Administration is also advocating for the inclusion of antitumor-targeted drugs in the national medical insurance catalog and has introduced several support policies, such as the 2009 “Opinions of the Central Committee of the Communist Party of China and the State Council on Deepening the Reform of the Medical and Health System” (Zhongfa [2009] No. 6) and the 2015 “Pilot Work Plan for Establishing a Drug Price Negotiation Mechanism (Draft for Comments)” (24) by the National Health Commission. The advent of targeted therapy has expanded the range of adverse reactions associated with cancer treatment. In addition to common side effects such as nausea, vomiting, constipation, diarrhea, and organ involvement, new skin toxicities present new challenges for clinical healthcare workers. The mechanism and risk factors of HFSR are yet to be clearly defined. Notably, the occurrence and severity of HFSR are dose-dependent. The continuous use of targeted drugs necessitates effective and scientific handling by healthcare workers, along with the active coping efforts of patients, to manage the symptoms effectively. Early preventive education for patients is crucial to reduce discomfort and prevent complications caused by symptoms.

The primary entries established in the present study include baseline and hand-foot skin assessment, skin erythema care, skin keratosis care, skin blister management, skin ulcer care, skin pain management, management of other accompanying symptoms, and hand-foot protection education, clearly categorizing each treatment measure. To prevent any omissions, a thorough search was conducted utilizing all targeted drug names as keywords. This approach aimed to investigate adverse reactions and identify potential derive intervention measures for HFSR. There is currently no specific nursing plan for HFSR symptom management and prevention, and clinical nursing interventions for HFSR are relatively few. Therefore, extensively soliciting and considering expert opinions is paramount.

A multidisciplinary team was formed during expert panel member selection, including experts from oncology, dermatology, pain management, and wound and stoma care. Such a collaborative multidisciplinary team can provide optimal care and support for cancer patients and their families. The selected experts for this study demonstrated a high level of familiarity with the content and a strong basis for their judgments, indicating good authority and representativeness. All experts provided suggestions and opinions in the two rounds of expert meetings, achieving a 100% positive response rate. The constructed plan is thus practical and valuable for healthcare practitioners.

### 4.2 Scientific and clinical applicability of the plan facilitates subsequent evidence translation research

With the advancement of molecular technology, targeted drugs have become suitable for the treatment of most tumors (25–27). However, these drugs are associated with a high incidence of skin adverse events, with HFSR being the most common and severe. This significantly impacts the physical and mental health, social well-being, and quality of life of patients, potentially leading to dose reduction or even discontinuation of the medication due to the body’s inability to tolerate these adverse reactions (28). So far, no study has systematically integrated HFSR symptom management and prevention. In addition, existing guidelines have only summarized all targeted therapy-induced skin toxicities without specificity, leading to scattered information on HFSR, which makes it difficult to draw comprehensive conclusions. Management and prevention of HFSR symptoms remain a pressing clinical issue. Introducing evidence into clinical settings and localizing, tailoring, organizing, and integrating this evidence is crucial for its clinical application (29).

Most of the included literature consisted of international guidelines and expert consensus. Given the differences in medical resources, local cultural backgrounds, and the specific needs and baseline characteristics of patients in China, expert meetings were conducted to adjust the content of the plan. For instance, since most hospitals in China do not have dedicated podiatrists and orthotists, the term was revised to “podiatry clinic doctors” in the program. In the original item 2.3.3.2, the suggestion to “apply sunscreen to the feet” was deleted, as patients with HFSR often use protective measures such as wearing thick cotton socks, making this recommendation unsuitable for Chinese patients. Additionally, references to medium- and high-potency corticosteroids in the original plan were changed to hydrocortisone, fluocinolone, and betamethasone propionate, which are frequently used in most hospitals in China. The current study strictly adhered to evidence-based principles, employing scientific and rigorous methods for evaluating the quality of literature. The entire procedure, comprising literature retrieval, evidence extraction, and indicator grading, was carried out independently and objectively by two individuals. This approach ensured the authenticity and reliability of the process.

Related studies indicate an imbalance between patients’ needs for coping with HFSR and the professional support provided by clinical healthcare personnel (30). The present study accounted for the needs of stakeholders, systematically listing the assessment and symptomatic treatment of each common HFSR symptom. Through the expert meeting method, the feasibility and appropriateness of each item were assessed, incorporating the opinions and suggestions from experts’ extensive clinical experience and research in the field. This approach narrows the gap between evidence and actual clinical practice, facilitating evidence translation.

## 5 Conclusion

In summary, nurses play a crucial role in managing patients’ skin toxic reactions and are ideally positioned to provide supportive care throughout the cancer treatment process. The goal of nursing management is to maintain treatment adherence and improve the quality of life of the patient through proactive and preemptive approaches. This study integrated more high-quality randomized controlled trials and relevant expert guidelines to develop the best management plan for patients with targeted therapy-induced HFSR. However, some of the included literature is relatively outdated, requiring careful discernment and judgment in content extraction. Further clinical application is needed to verify the effectiveness of this plan, and large-scale prospective randomized trials should be conducted to provide more insights into the management of HFSR.

## Data Availability

The raw data supporting the conclusions of this article will be made available by the authors, without undue reservation.
